# Voluntary Exercise Stabilizes Established Angiotensin II-Dependent Atherosclerosis in Mice through Systemic Anti-Inflammatory Effects

**DOI:** 10.1371/journal.pone.0143536

**Published:** 2015-11-24

**Authors:** Maxime Pellegrin, Jean-François Aubert, Karima Bouzourène, Catherine Amstutz, Lucia Mazzolai

**Affiliations:** Division of Angiology, University Hospital of Lausanne, Lausanne, Switzerland; Maastricht University, NETHERLANDS

## Abstract

We have previously demonstrated that exercise training prevents the development of Angiotensin (Ang) II-induced atherosclerosis and vulnerable plaques in Apolipoprotein E-deficient (ApoE^-/-^) mice. In this report, we investigated whether exercise attenuates progression and promotes stability in pre-established vulnerable lesions. To this end, ApoE^-/-^ mice with already established Ang II-mediated advanced and vulnerable lesions (2-kidney, 1-clip [2K1C] renovascular hypertension model), were subjected to sedentary (SED) or voluntary wheel running training (EXE) regimens for 4 weeks. Mean blood pressure and plasma renin activity did not significantly differ between the two groups, while total plasma cholesterol significantly decreased in 2K1C EXE mice. Aortic plaque size was significantly reduced by 63% in 2K1C EXE compared to SED mice. Plaque stability score was significantly higher in 2K1C EXE mice than in SED ones. Aortic ICAM-1 mRNA expression was significantly down-regulated following EXE. Moreover, EXE significantly down-regulated splenic pro-inflammatory cytokines IL-18, and IL-1β mRNA expression while increasing that of anti-inflammatory cytokine IL-4. Reduction in plasma IL-18 levels was also observed in response to EXE. There was no significant difference in aortic and splenic Th1/Th2 and M1/M2 polarization markers mRNA expression between the two groups. Our results indicate that voluntary EXE is effective in slowing progression and promoting stabilization of pre-existing Ang II-dependent vulnerable lesions by ameliorating systemic inflammatory state. Our findings support a therapeutic role for voluntary EXE in patients with established atherosclerosis.

## Introduction

Regular exercise training is an essential strategy for both primary and secondary cardiovascular disease prevention [1–5]. In a recent meta-analysis of prospective cohort studies, moderate and high levels of physical activity have been associated with a 12% and 27% relative risk reduction of coronary heart disease, respectively [5]. Along the same line, acute myocardial infarctions patients randomized to exercise-based cardiac rehabilitation had a 47% risk reduction for reinfarction, 36% for cardiac mortality, and 47% for all cause mortality, as recently revealed in a systemic review and meta-analysis of randomized controlled trials [3].

Exercise induces cardio-vascular benefits partly through its direct positive impact on atherosclerosis development and progression. Indeed, exercise is effective in decreasing carotid artery intima-media thickness in healthy asymptomatic subjects as well as in subjects with cardiovascular risk factors and/or disease [6,7]. Moreover, we and others have shown that exercise, including running as well as swimming, delays atherosclerosis progression, stabilizes, and reduces rupture of atherosclerotic plaque in Apolipoprotein E (ApoE) and low density lipoprotein receptor (LDLr) knockout mice [8–14]. Regression of experimental pre-existing atherosclerotic plaques has also been reported following exercise [15,16].

The renin-angiotensin system, and in particular its final product Angiotensin (Ang) II, plays a pivotal role in atherogenesis and plaque vulnerability [17]. We recently reported that exercise prevents the development of Angiotensin (Ang) II-induced advanced atherosclerosis and plaque vulnerability, using the 2-kiney, 1-clip ApoE^-/-^ mouse model [18]. In the present study, we investigated whether exercise has any effect on limiting progression of already established vulnerable Ang II-dependent atherosclerotic lesions.

## Methods

### Mouse model of Ang II-induced advanced and vulnerable plaque and voluntary running wheel exercise

Male and female C57BL/6J ApoE^-/-^ mice originally purchased from Charles River Laboratories (L’Arbresle, France) were used. Animals were housed in local animal facility under a 12-h light/dark cycle in a temperature-controlled environment and ad libitum access to normal chow diet (Kliba Nafag, Switzerland) and water throughout the study. All procedures were performed according to the Swiss Ethical Principles and Guidelines for Experiments on Animals, and with approval of local Institutional Animal Committee (“Service de la Consommation et des Affaires Vétérinaires du canton de Vaud”). All efforts were made to minimize suffering. At 12–14 weeks of age, mice underwent left renal artery clipping (2-kidney, 1-clip [2K1C] renovascular hypertension model) under anaesthesia to induce the formation of advanced and vulnerable Ang II-dependent lesions as previously described [18–20]. Briefly, mice were anesthetized by halothane inhalation (1% to 2% in oxygen), the left kidney was exposed and reduction in left renal perfusion was induced by placing a U-shaped stainless steel clip of 0.12 mm internal diameter around the renal artery. Renal perfusion reduction induces increased renin secretion from juxtaglomerular cells of clipped kidney leading to increased Ang II production and thus development of chronic Ang II-dependent systemic hypertension. Besides hypertension, 2K1C ApoE^-/-^ mice with high circulating Ang II develop advanced atherosclerotic lesions with vulnerable phenotype [18–20]. At 4 weeks, four mice were euthanatized to confirm the presence of such atherosclerotic lesions in aortic sinus (data not shown). Prior to surgery and for 2 days following surgery, mice were subcutaneously injected with Temgesic (0.01 mg/kg) for pain relief. Additionally, animals were treated with Dafalgan (200 mg/kg) via drinking water during 7 days following surgery.

The remaining 4-week 2K1C ApoE^-/-^ mice were randomly assigned to either a voluntary exercise group (EXE; n = 12 total, n = 7 males and n = 6 females) or a control sedentary group (SED; n = 14 total, n = 5 males and n = 9 females) for additional 4 weeks. EXE mice were individually housed and had free 24-h/day access to a running wheel (12 cm diameter).

### Hemodynamic parameters, plasma renin activity, and plasma total cholesterol measurements

At the end of the study (8 weeks after renal artery clipping), mean blood pressure (MBP) and heart rate (HR) were measured in conscious mice by using an intra-carotid catheter connected to a pressure transducer as described elsewhere [18–20]. Blood sample obtained through the catheter were used to determine plasma renin activity (PRA) by radioimmunoassay, and plasma total cholesterol levels using enzymatic methods [18–20]. Mice were then euthanized with sodium pentobarbital, tissues rapidly harvested, and processed for later analysis.

### En face analysis of atherosclerotic plaque extension

After fixation with 10% neutral formalin, aortas were removed from surrounding connective tissues, opened longitudinally, pinned on a petri dish with a black silicone surface, and stained with Oil red O to visualize atherosclerotic plaque. Pictures of stained aortas were taken with a digital camera (Coolpix, Nikon, Japan). Total aortic surface area and atherosclerotic plaque areas of each pinned aorta were measured using computer-assisted image analysis Leica Qwin software (Leica systems, Wetzler, Germany), and percentage of atherosclerotic lesion to total aortic surface area was calculated [19,20].

### Histologic, immunohistochemical, and morphometry analyses of atherosclerotic plaques

After fixation with 10% neutral formalin, hearts were paraffin embedded and cross-sectioned (3 μm) until reaching the aortic sinus. Aortic sinus sections were subsequently stained with Movat pentachrome or with Sirius red for lipid core quantification and total collagen plaque content assessment, respectively. Additionally, the relative plaque content of macrophages and smooth muscle (SM) cells was determined by immunostaining with primary anti-mouse Mac-2 (Cedarlane Labs, Ontario, Canada, dilution 1/200) and α-(SM) actin (gift of Dr M-L Piallat-Bochaton, University of Geneva, Switzerland, dilution 1/200) antibodies, respectively, followed by appropriate biotinylated secondary antibodies. Antibodies were revealed with a peroxidase-linked avidin-biotin detection system. Images of sections were captured with a digital camera, and specific staining was expressed as percentage of total cross-sectional plaque area using the Qwin software [18–20]. Histological plaque stability score was calculated as follows: (SM cell area + collagen area)/(macrophage area + lipid core area) [21,22].

### Real-time reverse transcription PCR and gene expression analysis

Total RNA from aortas and spleens was isolated (RNeasy Mini kit, Qiagen, Switzerland), and reverse transcribed into cDNA (iScriptT^™^ cDNA Synthesis Kit, Bio-Rad, Switzerland) following manufacturer’s instructions. Real-time RT-PCR (CFX96 Real-Time PCR detection system, Bio-Rad, Switzerland) using the iQ^™^ SYBER Green PCR Supermix was used to detect mRNA expression of VCAM-1, ICAM-1, IL-1β, IL-18, TNF-α, IL-6, IL-1ra, IL-4, IL-10, iNOS, Arg1, T-bet, GATA3 and 36B4. Primer sequences are listed in Table 1. Expression of target genes was normalized to the expression of the housekeeping 36B4 gene using the comparative Ct method. All data in EXE mice were expressed as fold change over SED mice, arbitrarily set at 1.

**Table 1:** Primers for quantitative real-time RT-PCR.

Gene	Forward primer	Reverse primer
VCAM-1	ATTTTCTGGGGAGGAAGTT	ACGTCAGAACAACCGAATCC
ICAM-1	AGCACCTCCCCACCTACTTT	AGCTTGCACGACCCTTCTAA
IL-1β	TCGCAGCAGCACATCAACAAG	TCCACGGGAAAGACACAGGTAG
IL-18	ACTTCTCCTGTTTGTTTGTG	TCTGGATACTGGGCTGTG
TNF-α	TAGCCAGGAGGGAGAACAGAAAC	CCAGTGAGTGAAAGGGACAGAAC
IL-6	GATGCTACCAAACTGGATATAATC	GGTCCTTAGCCACTCCT GTGTG
IL-1ra	ACAGTAGAAGGAGACAGAAG	GGTGGTAGAGCAGAAGAC
IL-4	TCAACCCCCAGCTAGTTGTC	TGTTCTTCGTTGCTGTGAGG
IL-10	GCACTACCAAAGCCACAAAGC	GTCAGTAAGAGCAGGCAGCATAG
iNOS	CCAAGCCCTCACCTACTTCC	CTCTGAGGGCTGACACAAGG
Arg1	CTCCAAGCCAAAGTCCTTAGAG	AGGAGGTGTCATTAGGGACATC
T-bet	AACCAGTATCCTGTTCCCAGC	TGTCGCCACTGGAAGGATAG
GATA3	CAGAACCGGCCCCTTATCA	CATTAGCGTTCCTCCTCCAGA
36B4	ATGGGTACAAGCGCGTCCTG	GCCTTGACCTTTTCAGTAAG

### Measurements of circulating inflammatory cytokines

Plama levels of IL-1β, IL-18 and IL-4 were quantified using commercial mouse enzyme-linked immunosorbent assay (ELISA) kits (R&D Systems, MBL-Medical and Biological Laboratories, and eBioscience), respectively, following manufacturer instructions.

### Statistical analysis

No significant differences between sexes were found and results were averaged. Data are presented as mean ± SD or as interquartile ranges ± minimum and maximum values. Statistical analysis was performed using the unpaired student’s t-test. Analysis of body weight (BW) and running distance were evaluated using one-way ANOVA followed by Tukey’s multiple comparisons test. A value of P<0.05 was considered statistically significant.

## Results

### Effect of voluntary exercise on physiological parameters

Physiological parameters of 8-week 2K1C ApoE^-/-^ SED and EXE mice are presented in Table 2. MBP, HR, and PRA did not significantly differ between the two groups. Total cholesterol plasma levels were significantly reduced by 32% in EXE mice (P<0.05 versus SED). There was no significant difference in initial BW (i.e. before EXE) between groups either in male (30.0±3.0 for SED versus 27.6±2.1 for EXE) or in female mice (22.3±1.1 versus 22.5±1.6). Similarly, BW did not significantly differ between SED and EXE male (32.5±2.8 versus 30.0±1.4 for EX) and female mice (23.3±1.4 versus 24.1±1.0) at end of study. Average daily running distance covered by EXE mice was 7.3±0.9km. Weekly running distance increased significantly during the first three weeks (6.3±0.4km versus 7.6±0.2km versus 8.4±0.4km, respectively; P<0.05), stabilizing at 6.8±0.6km during the last week.

**Table 2:** Comparison of physiological parameters in 8-week 2K1C ApoE^-/-^ SED and EXE mice.

	SED	EXE
MBP (mmHg)	143±9.0	147±12
HR (bpm)	602±76	604±47
PRA (ng/ml per h)	8.4±3.7	8.9±3.6
Total plasma cholesterol levels (mg/dL)	576±166	394±54*

MBP: mean blood pressure; HR: heart rate; PRA: plasma renin activity.

*P<0.05 versus SED.

N = 13–14 mice per group for MBP, HR and PRA, and N = 6 mice per group for total cholesterol levels.

Data are presented as mean ± SD.

### Effect of voluntary exercise on progression and phenotype of Ang II-dependent pre-existing atherosclerotic lesions

**Fig 1:**
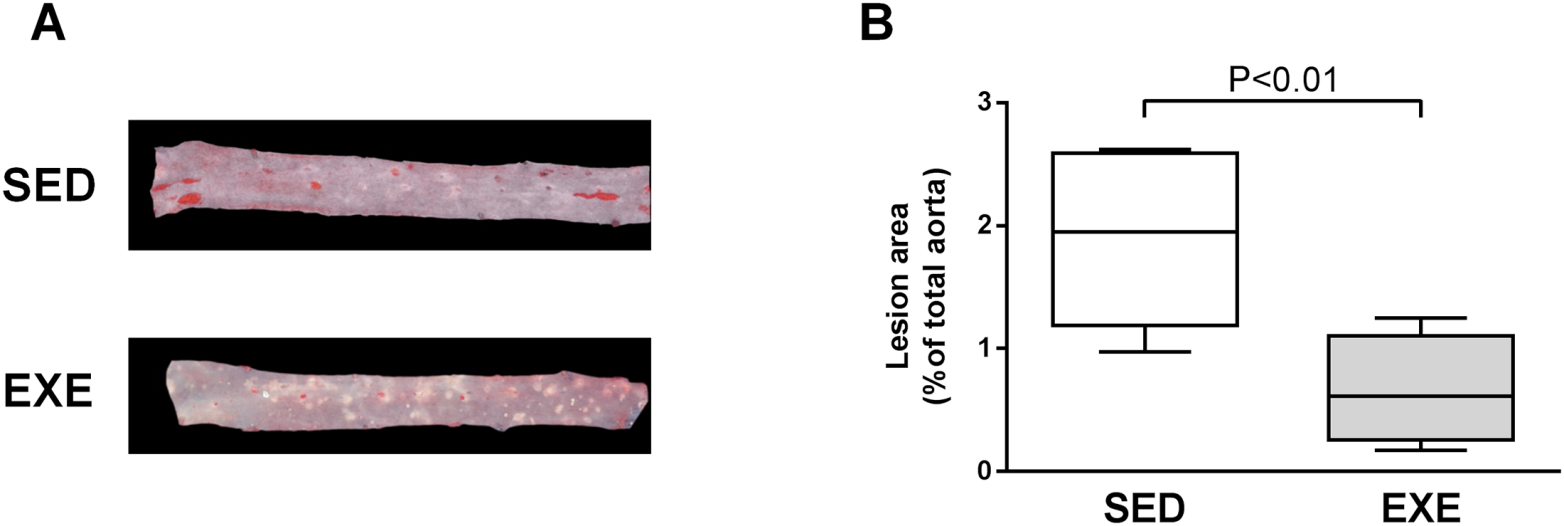
Comparison of atherosclerotic plaque extension among 8-week 2K1C ApoE^-/-^SED and EXE mice. A, Representative photomicrographs of en face aortas stained with Oil red O. B, Quantitative morphometric analysis of Oild red O-stained lesions as percentage of total aorta surface. N = 7 mice per group. Box plots display interquartile ranges, whiskers indicate minimum and maximum values.

Analysis of Oil Red O-stained aortas revealed a significant 63% reduction in atherosclerotic lesions area in EXE compared to SED mice (1.88±0.71% versus 0.70±0.16%, P<0.01, Fig 1).

**Fig 2:**
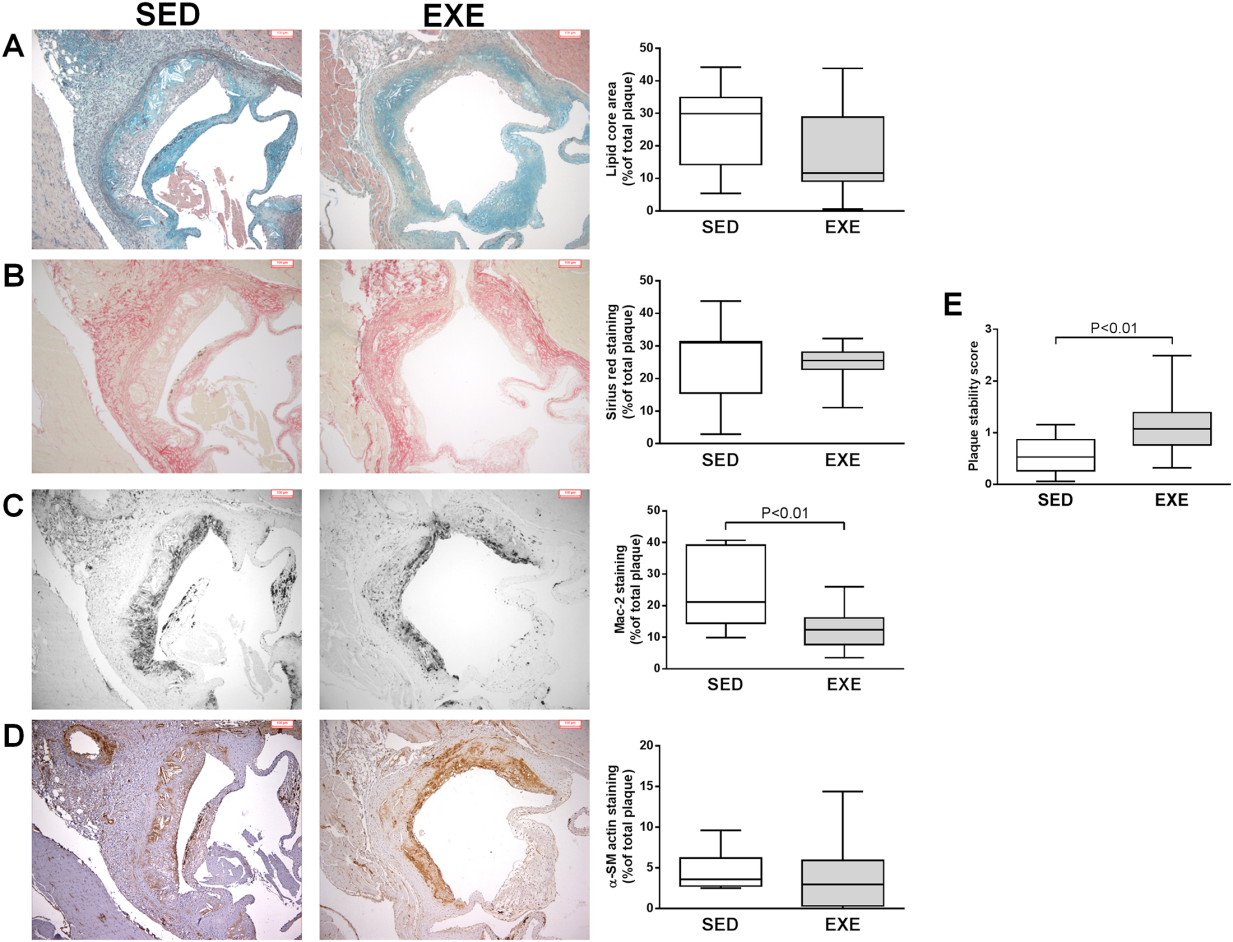
Comparison of atherosclerotic plaques phenotype among 8-week 2K1C ApoE^-/-^SED and EXE mice. Representative light microscopic photomicrographs and quantitative morphometric analysis of Movat pentachrome staining of lipid core areas (A), Sirius red staining of total collagen (B), anti-Mac-2 staining of infiltrated macrophages (C), and anti-α-SM actin staining of SM cells in atherosclerotic plaques from the aortic sinus. Results are expressed as percentage of staining area to total plaque area. (D), Plaque stability score calculated with the formula described in “methods” section. N = 9 mice in SED group; N = 14 mice in EXE group. Box plots display interquartile ranges, whiskers indicate minimum and maximum values.

Plaque lipid core area, and collagen content did not differ among the two groups of mice (Lipid core size: 25.5±12.8% in SED versus 17.1±13.2% in EXE, P = 0.15, Fig 2A; collagen plaque content: 25.9±12.3% in SED versus 24.5±6.2% in EXE mice, P = 0.72, Fig 2B).

On the contrary, macrophage plaque content was significantly reduced by 53% in EXE mice compared to SED ones (26.0±12.4% versus 12.2±6.0%, P<0.01, Fig 2C). No significant difference in SM cells plaque content was observed between SED (4.5±2.6%) and EXE animals (4.3±4.9%) (Fig 2D).

Plaque stability score is shown in Fig 2E. EXE mice showed a significantly 98% higher score than SED mice (1.13±0.52 versus 0.57±0.35, P<0.01), indicating a more stable plaque phenotype.

### Effect of voluntary exercise on local and systemic pro- and anti-inflammatory mediators

**Fig 3:**
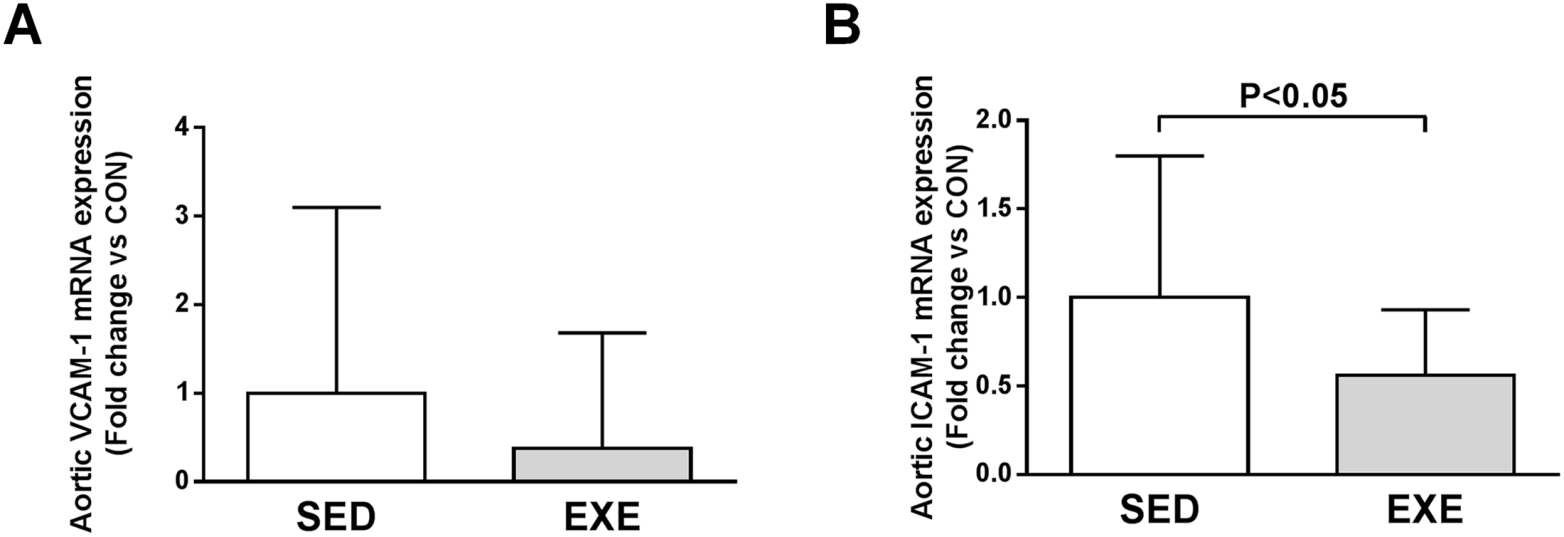
Comparison of local endothelial adhesion molecules expression among 8-week 2K1C ApoE^-/-^ SED and EXE mice. Aortic mRNA expression of VCAM-1 (A) and ICAM-1 (B) measured by quantitative real-time RT-PCR. Data are expressed as fold change ± SD compared to SED (set at 1) after normalization to the 36B4 housekeeping gene.

Quantitative RT-PCR analysis revealed that expression of VCAM-1 and ICAM-1 in atherosclerotic aortic tissue decreased in EXE compared to SED mice (0.38-fold, P = 0.085 and 0.56-fold, P<0.05, respectively, Fig 3).

**Fig 4:**
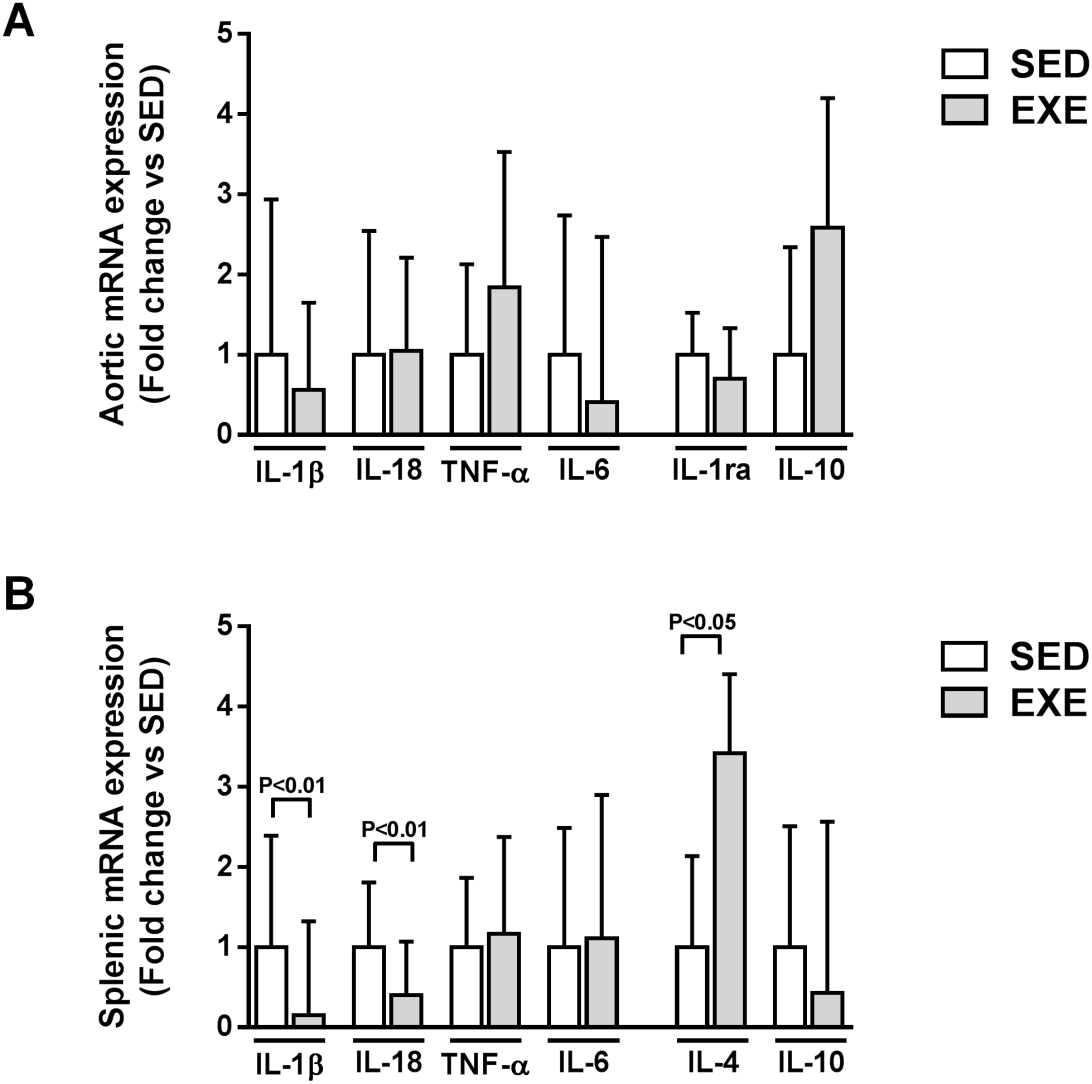
Comparison of local and systemic cytokines expression among 8-week 2K1C ApoE^-/-^ SED and EXE mice. Aortic (A) and splenic (B) mRNA expression of pro-inflammatory cytokines IL-1β, IL-18, TNF-α and IL-6 cytokines and anti-inflammatory cytokines IL-1ra, IL-4 and/or IL-10 measured by quantitative real-time PCR. N = 6 mice in SED group; N = 7 mice in EXE group. Data are expressed as fold change ± SD compared to SED (set at 1) after normalization to the 36B4 housekeeping gene.

As shown in Fig 4A, no significant difference in aortic expression of pro-inflammatory (IL-1β, IL-18, TNF-α, and IL-6) or anti-inflammatory cytokines (IL-1ra and IL-10) was observed between the two groups. Interestingly, splenic IL-1β and IL-18 expression significantly decreased (0.15- and 0.40-fold respectively, P<0.01), while IL-4 expression significantly increased (3.4-fold, P<0.05) following EXE (Fig 4B). EXE did not change TNF-α, IL-6 or IL-10 splenic expression (Fig 4B).

Consistent with RT-PCR results, plasma IL-18 concentration decreased in EXE mice compared to SED mice (148.1 ± 52.1 pg/ml versus 198.4 ± 59.2, p = 0.09, n = 7–9 mice per group). IL-1β and IL-4 levels were not detectable in plasma in either SED or EXE mice.

### Effect of voluntary exercise on local and systemic pro- and anti-inflammatory macrophages, and CD4^+^ T helper cells

**Fig 5:**
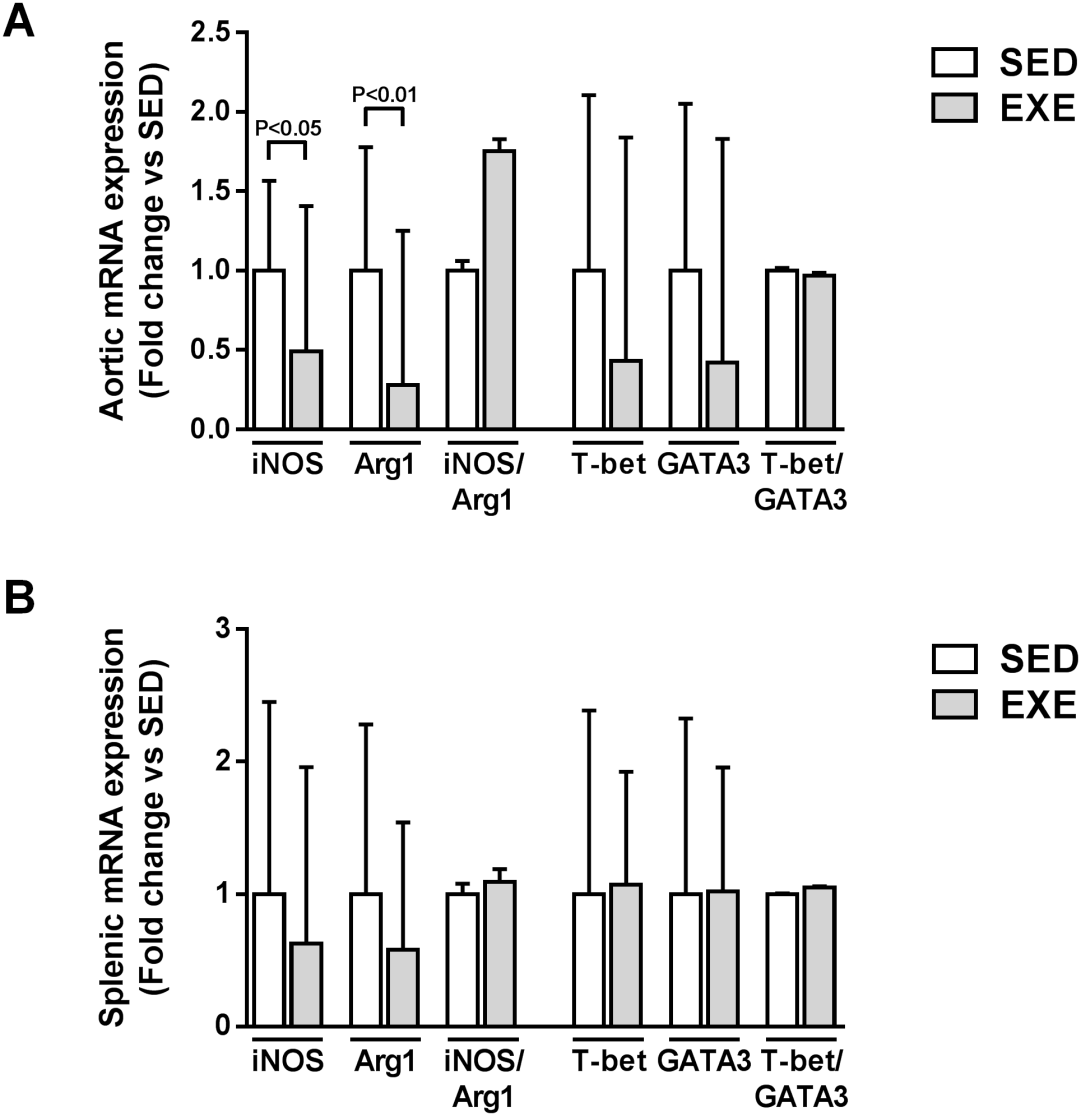
Comparison of local and systemic CD4^+^ T helper cells and macrophage polarization markers expression among 8-week 2K1C ApoE^-/-^ SED and EXE mice. Aortic (A) and splenic (B) mRNA expression of pro-inflammatory M1 macrophages (iNOS) and Th1 cells (T-bet) and anti-inflammatory M2 macrophages (Arg1) and Th2 cells (GATA3) measured by quantitative real-time PCR. Fold change ratios of iNOS to Arg1 (M1/M2 balance marker) and of T-bet to GATA3 (Th1/Th2 cells balance marker) were calculated. N = 6 mice in SED group; N = 7 mice in EXE group. Data are expressed as fold change ± SD compared to SED (set at 1) after normalization to the 36B4 housekeeping gene.

EXE mice showed significant reduction in aortic iNOS (pro-inflammatory M1 macrophage marker), and Arg1 (anti-inflammatory M2 macrophage marker) expression (0.49-fold, P<0.05 and 0.28-fold, P<0.01, respectively, Fig 5A). As a result, there was no significant difference in iNOS/Arg1 expression ratio (M1/M2 macrophage balance marker) (Fig 5A). No significant difference in either T-bet (pro-inflammatory Th1 marker), GATA3 (anti-inflammatory Th2 marker), or T-bet/GATA3 ratio (Th1/Th2 balance marker) expression was observed between SED and EXE mice (Fig 5A).

As shown in Fig 5B, there was no significant difference in iNOS, GATA3, iNOS/Arg1, T-bet, GATA3 and T-bet/GATA3 splenic expression between the two groups.

## Discussion

To our knowledge, the present study is the first one to examine whether EXE is effective in limiting progression of Ang II-induced pre-existing vulnerable atherosclerotic lesions. Main findings can be summarized as follows: (i) Voluntary EXE limited further ATS progression, and promoted stability of established Ang II-dependent plaques; (ii) Voluntary EXE reduced aortic VCAM-1 and ICAM-1 expression; (iii) Voluntary EXE reduced splenic expression of pro-inflammatory cytokines (IL-1β and IL-18), while increasing that of anti-inflammatory IL-4; (iv) Voluntary EXE did not modulate aortic and splenic expression of Th1/Th2, and M1/M2 polarization markers; finally, (v) Voluntary EXE reduced total plasma cholesterol level, but not blood pressure, and plasma renin activity.

Atherosclerotic plaque destabilization and subsequent rupture is the main pathologic mechanism responsible for a majority of cardiovascular events. Herein, we showed that voluntary EXE stabilized pre-existing Ang II-dependent plaques mainly by reducing lesional macrophage infiltration. This result corroborates earlier reports both in clipped and non-clipped ApoE^-/-^, and/or LDLr^-/-^ mice [9,10,12,13,16]. The reduction in macrophage plaque content following EXE correlated with a vascular reduction in endothelial adhesion molecules ICAM-1 and VCAM-1 (although not significant) expression. Since these molecules are known to participate in atherogenesis by promoting monocytes recruitment, which differentiate to macrophages in the arterial intima, we propose that the observed decrease in their expression constitute a mechanisms of action by which voluntary EXE prevents plaque inflammation.

Cytokines such as IL-1β, IL-18, IL-6 and TNF-α are key mediators in chronic vascular inflammatory response underlying several aspects of atherosclerosis and cardiovascular disease [23–26]. Numerous animal studies demonstrated that vascular decreased expression of such pro-inflammatory cytokines, in response to pharmacological treatments, is associated with atherosclerosis burden prevention [27–29]. In the present study, voluntary EXE failed to modulate vascular expression of the above-mentioned cytokines. Moreover, no difference in vascular anti-inflammatory/atherosclerotic cytokines IL-1ra and IL-10 was observed between EXE and SED mice. These results indicate that voluntary EXE has no positive impact on local inflammation in our model. In accordance with these findings, we previously found no effect of 4-week forced treadmill EXE on local vascular IL-6, IL-1β, TNF-α IL-18, IL-10 and IL-1ra expression despite Ang II-dependent vulnerable plaque prevention in 4-week 2K1C ApoE^-/-^ mice (unpublished data). Thus, modulation of vascular inflammation does not seem to be a mechanism of action by which voluntary EXE promotes stabilization of established Ang II-dependent plaque.

Atherosclerosis is a systemic disease and there are compelling clinical and experimental evidences showing that EXE has anti-inflammatory systemic effects [30,31]. For example, in patients with stable coronary artery disease, circulating C-reactive protein, and IL-6 levels were significantly reduced after regular EXE by 41 and 18%, respectively [32]. In a recent randomized clinical trial, Ribeiro et al. also demonstrated increased circulating IL-10 levels after an 8-week aerobic EXE program in post-myocardial infarction patients [33]. Along the same line, a reduction in systemic IL-1, IL-6 and increased in IL-10 levels was reported in coronary heart disease patients in response to a 12-week aerobic EXE training program [34]. Serum pro-inflammatory cytokines reduction in response to EXE has also been reported in ApoE^-/-^ mice with advanced atherosclerosis [8,9]. Based on these considerations, we hypothesized that voluntary EXE would ameliorate the systemic inflammatory status of our mice. Interestingly, voluntary EXE favorably modified systemic balance of pro- and anti-inflammatory cytokines, as evidenced by reduced IL-1β, IL-18, and increased IL-4 expression in spleen tissue. Reduction in plasma IL-18 levels was also observed in response to voluntary EXE. Taken together, our data suggest that in our mouse model voluntary EXE exerts atheroprotection through systemic rather than local anti-inflammatory properties.

Immune cells, both from innate and adaptive immunity, are present throughout all stages of atherosclerotic lesion development. Lesions’ innate immune cells are predominantly monocytes/macrophages while adaptive ones are mostly CD4^+^ T cells [35,36]. Naive CD4^+^ T cells have the ability to differentiate into various T helper subtypes. These include in particular Th1-cell lineage which are pro-atherogenic via the secretion typically of IFN-γ and TNF-α, and Th2-cell lineage secreting IL-4, IL-10 and IL-13 whom their role in atherosclerosis remains controversial, including Th1 and Th2 cells [35,36]. Like CD4^+^ T cells, macrophages can alter their phenotypes and functions accordingly in response to change in the microenvironment. In the setting of atherosclerosis, the concept of macrophage polarization is increasing given recent work showing that different stages in the progression of atherosclerosis are associated with the presence of distinct macrophage subtype (i.e. pro-inflammatory/atherosclerotic M1 or classically activated versus anti-inflammatory/atherosclerotic M2 or alternatively activated macrophages) [37]. To further understand cellular mechanisms underlying our observations, we examined expression of genes associated with Th1- and Th2-polarization (T-bet and GATA3, respectively) as well as M1- and M2-polarization (iNOS and Arg1, respectively) in aortic and spleen tissues. Neither Th1/Th2 cells, nor M1/M2-associated gene expression were modulated by voluntary EXE. These findings corroborate recent investigations from our group showing no effect of 4-week forced treadmill EXE in 2K1C ApoE^-/-^ mice on M1 and M2 polarization (unpublished data).

In conclusion, we showed that voluntary EXE is an effective therapeutic strategy to slow down progression and promote stabilization of pre-existing Ang II-mediated atherosclerotic lesions. As proposed mechanisms, voluntary EXE decreases vascular adhesion molecules VCAM-1 and ICAM-1, and ameliorates systemic inflammatory profile by decreasing pro-inflammatory IL-1β, IL-18 cytokines, and increasing anti-inflammatory IL-4 cytokine. Our findings strongly highlight clinical relevance of voluntary regular EX for patients with established atherosclerosis.
